# The first cardioverter defibrillator implanted in Central Africa

**DOI:** 10.11604/pamj.2016.23.115.3822

**Published:** 2016-03-17

**Authors:** Tantchou Tchoumi Jacques Cabral, Appolonia Budzee, Gianfranco Butera

**Affiliations:** 1 St Elizabeth Catholic General Hospital, Cardiac Centre, Kumbo, Cameroon; 2 Policlinico San Donato IRCCS, San Donato Milanese, Milan, Italy

**Keywords:** Implantable cardioverter defibrillator, hypertrophic cardiomyopathy, sudden death

## Abstract

Sudden cardiac deaths, which account for approximately 350 000 deaths each year, is a major health care problem. Antiarrhythmic drugs have not been reliable in preventing sudden cardiac death. Although β-blockers, angiotensin-converting enzyme inhibitors, and revascularization play a role in prevention of sudden cardiac death, the development and subsequent refinement of the implantable cardioverter-defibrillator has made the most important contribution to its management. We report the first documented implantation of a cardioverter defibrillator in central, eastern and western Africa.

## Editorial

Sub-Saharan Africa is dominated by diseases caused by poverty. HIV/AIDS affects 28.5 out of a total of 600 million in the region. As a result of progressive urbanization and westernization of their lifestyle, developing Countries are now undergoing an epidemiological transition [1]. These changes are leading to a new epidemiological situation in the world with a decline in infectious diseases and emergence of cardiovascular diseases [2]. Hypertrophic cardiomyopathy (HCM) is the most common genetically inherited cardiac disorder with an estimated prevalence of 1 in 500 in the general population [3]. Sudden cardiac death (SCD) is the most devastating consequence of the disease and HCM is now recognized as most frequent cause of sudden cardiac-related deaths in pre-adolescent and adolescent children, as well as young athletes [4].

Because most HCM patients at high risk for sudden death are young, with no or only mild symptoms and preserved systolic function, prevention of sudden death by the ICD may prolong life substantially in this disease and could offer a normal or near-normal life expectancy to many patients [5, 6]. Indeed, recent investigations have shown that the ICD is effective in preventing sudden death in HCM [7]. South Africa is the only Country in sub-Saharan Africa in which implantable cardiovertor defibrillators (ICDs) are implanted (0.8/million in 2001) [8].

For the first time in the rest of Africa, we document the first implantation of an ICD in the english literature in a young patient with HCM who had episodes of syncope of unknown etiology. The case was diagnosed several years ago; “medication was prescribed, but I was not better,” said Mr. A, who benefited from the procedure. The patient was asked to come to the Cardiac Centre for better management and treatment. The echocardiogram showed a very hypertrophied septum 38mm, and posterior wall 37mm, without left ventricular outflow obstruction and mild diastolic impairment. The patient was prescribed beta blockers and advised to consider the implantable cardioverter defibrillator. The crises of syncope and palpitation continued and the best way to proceed was the ICD implantation. The implantation (Figure 1,2) was done with success and the patient was discharged three days after the procedure.

Planted in the 75 year-old St. Elizabeth Catholic Hospital, the Shisong Cardiac Centre is the fruit of efforts between the Tertiary Sisters of St. Francis, Cuore Fratello and Bambini Cardiopatici nel Mondo Association of San Donato, Milan in Italy. This is the only cardio-surgical Center in Central / West Africa, equipped with ultra modern health technologies and prepared to offer diverse services: cardiology, including diagnosis and treatment of congenital heart defects, coronary artery disease, valvular heart disease and electrophysiology [9].

It manages non-invasive cardiology; that is, diagnostic testing for patients with suspected cardiac problems through tests such as electrocardiography, holter, stress test, three-dimensional, color, pulsed and continuous Doppler-echocardiography. It also offers both diagnostic and interventional catheterization in a heamodynamic laboratory, implantation of permanent mono- and bicameral pace makers and defibrillator as well as open-heart surgeries with extracorporeal circulation.

Numerous surgical teams travel to underdeveloped Countries to perform surgery each year and train the local surgeon and ancillary personnel as best they can. However, in most of such cases, the surgical teams are present at those sites for no more than one week per year, leaving the local population and surgeons to struggle themselves for the remainder of the year. Thanks to the dynamic skill in organization and management of the executive board, the Shisong Cardiac Centre has a resident cardiac surgeon. Almost three hundred cases have been operated with extracorporeal circulation, 54 pacemakers have been implanted (forty single chamber and 14 double chamber), interventional and diagnostic catheterizations were performed in 350 patients.

The first implantation of an ICD is opening a new page in the management of cases with life threatening arrhythmias in Africa and in Cameroon.
